# Developing and Demonstrating the Viability and Availability of the Multilevel Implementation Strategy for Syncope Optimal Care Through Engagement (MISSION) Syncope App: Evidence-Based Clinical Decision Support Tool

**DOI:** 10.2196/25192

**Published:** 2021-11-16

**Authors:** Shiraz Amin, Vedant Gupta, Gaixin Du, Colleen McMullen, Matthew Sirrine, Mark V Williams, Susan S Smyth, Romil Chadha, Seth Stearley, Jing Li

**Affiliations:** 1 Performance Analytics Center of Excellence University of Kentucky HealthCare Lexington, KY United States; 2 Department of Cardiovascular Medicine University of Kentucky HealthCare Lexington, KY United States; 3 Center for Health Services Research University of Kentucky Lexington, KY United States; 4 Gill Heart & Vascular Institute University of Kentucky HealthCare Lexington, KY United States; 5 Division of Hospital Medicine Washington University School of Medicine St. Louis, MO United States; 6 College of Medicine University of Arkansas for Medical Sciences Little Rock, AR United States; 7 Division of Hospital Medicine University of Kentucky HealthCare Lexington, KY United States; 8 Department of Emergency Medicine University of Kentucky HealthCare Lexington, KY United States; 9 Department of Medicine Washington University School of Medicine St. Louis, MO United States

**Keywords:** cardiology, medical diagnosis, medicine, mobile applications, prognostics and health, syncope

## Abstract

**Background:**

Syncope evaluation and management is associated with testing overuse and unnecessary hospitalizations. The 2017 American College of Cardiology/American Heart Association (ACC/AHA) Syncope Guideline aims to standardize clinical practice and reduce unnecessary services. The use of clinical decision support (CDS) tools offers the potential to successfully implement evidence-based clinical guidelines. However, CDS tools that provide an evidence-based differential diagnosis (DDx) of syncope at the point of care are currently lacking.

**Objective:**

With input from diverse health systems, we developed and demonstrated the viability of a mobile app, the Multilevel Implementation Strategy for Syncope optImal care thrOugh eNgagement (MISSION) Syncope, as a CDS tool for syncope diagnosis and prognosis.

**Methods:**

Development of the app had three main goals: (1) reliable generation of an accurate DDx, (2) incorporation of an evidence-based clinical risk tool for prognosis, and (3) user-based design and technical development. To generate a DDx that incorporated assessment recommendations, we reviewed guidelines and the literature to determine clinical assessment questions (variables) and likelihood ratios (LHRs) for each variable in predicting etiology. The creation and validation of the app diagnosis occurred through an iterative clinician review and application to actual clinical cases. The review of available risk score calculators focused on identifying an easily applied and valid evidence-based clinical risk stratification tool. The review and decision-making factors included characteristics of the original study, clinical variables, and validation studies. App design and development relied on user-centered design principles. We used observations of the emergency department workflow, storyboard demonstration, multiple mock review sessions, and beta-testing to optimize functionality and usability.

**Results:**

The MISSION Syncope app is consistent with guideline recommendations on evidence-based practice (EBP), and its user interface (UI) reflects steps in a real-world patient evaluation: assessment, DDx, risk stratification, and recommendations. The app provides flexible clinical decision making, while emphasizing a care continuum; it generates recommendations for diagnosis and prognosis based on user input. The DDx in the app is deemed a pragmatic model that more closely aligns with real-world clinical practice and was validated using actual clinical cases. The beta-testing of the app demonstrated well-accepted functionality and usability of this syncope CDS tool.

**Conclusions:**

The MISSION Syncope app development integrated the current literature and clinical expertise to provide an evidence-based DDx, a prognosis using a validated scoring system, and recommendations based on clinical guidelines. This app demonstrates the importance of using research literature in the development of a CDS tool and applying clinical experience to fill the gaps in available research. It is essential for a successful app to be deliberate in pursuing a practical clinical model instead of striving for a perfect mathematical model, given available published evidence. This hybrid methodology can be applied to similar CDS tool development.

## Introduction

Syncope is a common yet complex presenting symptom that requires thoughtful and efficient evaluation to determine the etiology of the patient’s loss of consciousness (LOC). Estimates indicate that one-half of all Americans will experience syncope during their lives, with recurrence rates as high as 13.5% [1]. The prognosis of a patient with syncope depends on the etiology and other potential underlying medical conditions. Although vasovagal reflex–mediated syncope and orthostatic hypotension are the two most common types with benign courses, a cardiac or neurologic etiology of syncope is associated with significantly higher rates of morbidity and mortality [2]. The major challenge in the evaluation of patients with syncope is that most patients are asymptomatic at the time of their presentation. Because of concerns that patients presenting with syncope are at risk for an impending catastrophic event, the overuse and inappropriate use of testing and hospital admission are common [1]. Substantial published research, including our team’s work, documents the current practice of underutilization of efficient tests, overutilization of unnecessary tests, overexpenditure associated with syncope management, and heightened risk to patients due to unnecessary tests and hospitalizations [3-6]. Aiming to provide guidance on optimizing the evaluation and management of syncope, a collaboration of the American College of Emergency Physicians (ACEP), the Society for Academic Emergency Medicine (SAEM), the American College of Cardiology (ACC), the American Heart Association (AHA), and the Heart Rhythm Society (HRS) issued a *Guideline for the Evaluation and Management of Patients With Syncope* in 2017 (ie, 2017 Syncope Guideline) [7]. However, studies have found that awareness and implementation of the 2017 Syncope Guideline remain low, and current practice in the evaluation and management of syncope substantially deviates from clinical practice guidelines (CPGs) [3,8].

The use of clinical decision support (CDS) tools offers the potential to successfully implement evidence-based clinical guidelines. To assist clinicians in assessing patient risk, several syncope risk stratification calculators have been developed over the past 20 years, each with slightly different predicted clinical outcomes within various time frames [9-13]. Although risk calculators are useful, these existing tools for syncope function as medical risk calculators and do not provide any diagnosis or follow-up recommendations. CDS tools that provide an evidence-based differential diagnosis (DDx) of syncope at the point of care are currently lacking. A shared decision-making (SDM) tool for low-to-intermediate-risk patients with unexplained syncope who present to an academic emergency department (ED) in the United States is being developed but is still in the feasibility stage [14]. By incorporating CPGs and clinicians’ input, CDS tools hold great potential for improving evaluation, diagnosis, care delivery, and, ultimately, outcomes in patients presenting with syncopal symptoms.

**Figure 1:**
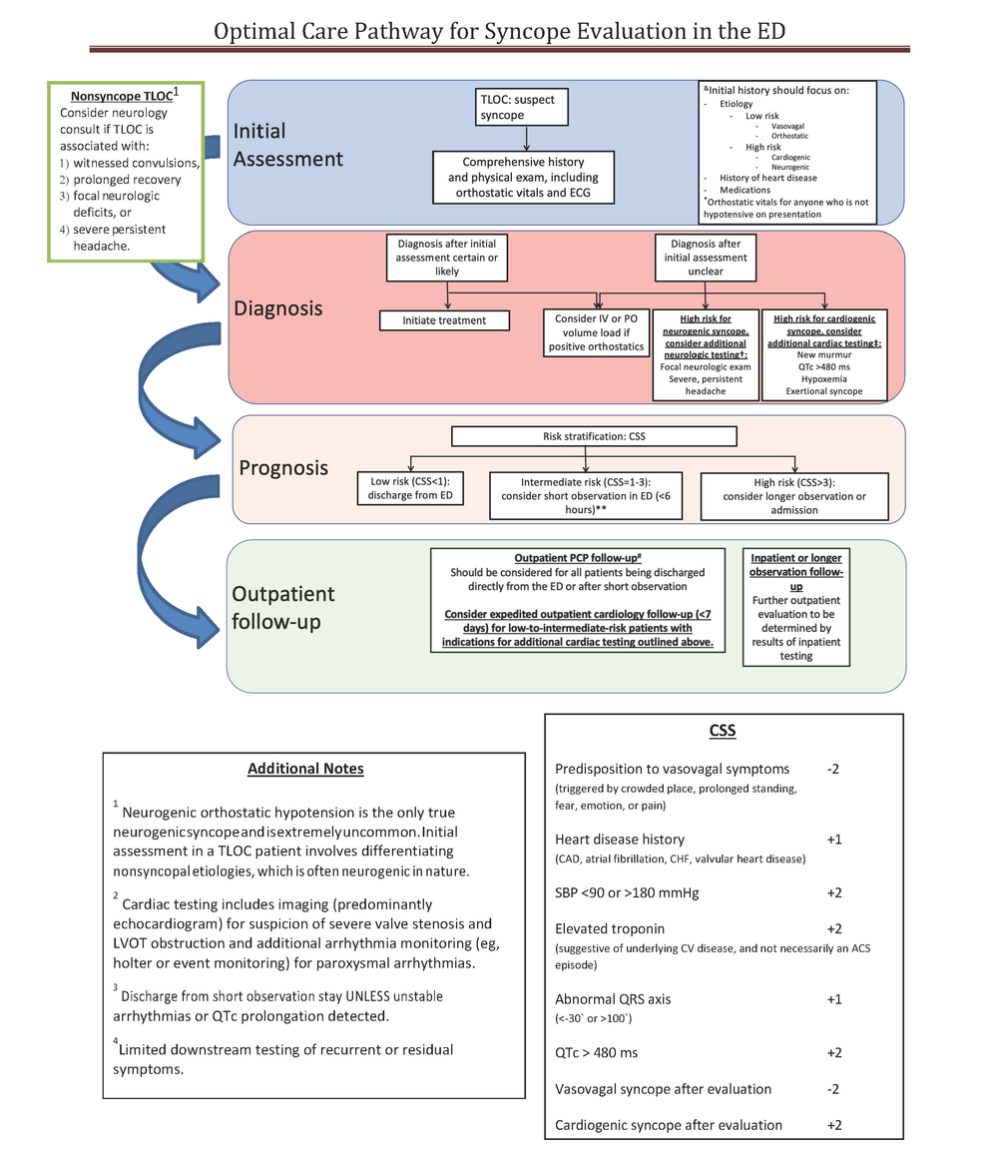
The MISSION Syncope OptimalCare Pathway. This is the pathway to be used by clinicians to adapt and implement EBPs, engage interdisciplinary expertise, and facilitate care delivery that reduces variability, improves quality, and lowers cost. ACS: acute coronary syndrome; CAD: coronary artery disease; CHF: congestive heart failure; CSS: Canadian Syncope Score; CV, cardiovascular; EBP: evidence-based practice; ECG: electrocardiogram; ED: emergency department; IV, intravenous; LVOT: left ventricular outflow tract; MISSION: Multilevel Implementation Strategy for Syncope optImal care thrOugh eNgagement; PCP: primary care provider; PO: per os; SBP: systolic blood pressure; TLOC: transient loss of consciousness.

Developing a multicomponent, Multilevel Implementation Strategy for Syncope optImal care thrOugh eNgagement [MISSION]) is a multisystem implementation study aiming to adopt, adapt, and implement evidence-based practices (EBPs); engage interdisciplinary expertise; and facilitate care delivery that reduces variability, improves quality, and lowers cost. With input from diverse health systems, our study team developed the MISSION Syncope OptimalCare Pathway (Figure 1) based on the 2017 Syncope Guideline. The MISSION Syncope mobile app was designed to be a practical tool for the implementation of the MISSION Syncope OptimalCare Pathway.

The objectives of this study were to develop and demonstrate the viability of the MISSION Syncope app as a CDS tool for syncope diagnosis and prognosis that walks users through clinical assessment in a clear and concise manner consistent with EBPs and to provide recommendations based on input from the user.

## Methods

The development of the app was a multistep process that included (1) reliable generation of an accurate DDx, (2) incorporation of an evidence-based clinical risk tool for prognosis, and (3) user-based design and technical development. The internal review board at the University of Kentucky approved this study.

### DDx

This process was guided by integrating the current literature and clinical expertise. Current guidelines recommend an initial assessment based on a comprehensive history, physical exam, orthostatic vital signs, basic laboratory findings, and a resting electrocardiogram (ECG) [7]. These recommendations are broad and incorporate a wide range of data points and clinical indicators that complicate meaningful implementation in an app. Therefore, it was first necessary to determine relevant and proven clinical indicators that are critical in assisting diagnosis and prognosis assessment.

A literature search using PubMed and Google Scholar databases consisted of the search terms “syncope,” “orthostatic hypertension,” “neurogenic syncope,” “vasovagal syncope,” and “cardiogenic syncope.” The search was limited to studies on humans. In addition, we analyzed the reference lists of identified articles to confirm we had not missed any relevant literature. The final literature search was performed on February 20, 2020. Based on the literature review, we compiled an initial list of variables; these were then reviewed by an interdisciplinary team of clinicians and consolidated into a final list. This literature review focused on the quality of study, identification of common predictors, and consolidation of study results through subject expert review and feedback. Some variables were consolidated to establish consistency and minimize different cutoffs (eg, an appropriate QT interval, ie, the time from the start of the Q wave to the end of the T wave, in different studies had varying optimal cutoff values). Focusing on app usability, the goal was to have 15-20 questions (ie, variables). This required consolidation if questions were deemed to assess a similar clinical condition and had comparable odds or likelihood ratios (LHRs). For example, a history of heart disease generically, ischemic heart disease, and atrial fibrillation were reported in separate studies with LHRs ranging from 2.4 to 7.3 [15,16]. Given the collinearity of these diseases associated with syncope, the strength of the studies, and the relative LHRs of other variables, a decision was made to consolidate these into a single question and associate the LHR from the most appropriate study.

Based on the clinical metrics identified in the literature review, we defined the inputs for a statistical model to provide an evidence-based DDx. This was done with an interdisciplinary team consisting of app developers, subject experts, and a statistician. To determine a diagnosis using a statistical model, we needed to identify the LHR for each question. This process was completed based on the quality of published research studies and subject expert (author VG) review, with special attention to variables without an LHR, a highly varying LHR, or an LHR that seemed discordant from other clinical data. This consideration included assessing the quality of original study data, sample size, patient population studied, and how the study applied to a general adult patient population.

We used the logistic regression model to calculate posttest log-odds of each cause of syncope (vasovagal, orthostatic, or cardiogenic) and included neurogenic LOC (eg, seizure) as the highest-risk nonsyncopal etiology. We defined binary logistic regression models for each etiology based on the LHRs we identified from the literature review. The use of LHRs can be beneficial in diagnosing individual patients [17]. Mathematically, a binary logistic model has a dependent variable with two possible values, which we defined as yes or no for each etiology. In the logistic model, the log-odds for the value labeled “yes” is a linear combination of one or more independent variables (Equation 1). The log-odds can then be transformed into a probability for a “yes” for each etiology (Equation 2).

For the model to work, we needed to define the *β_i_* parameters for the model. Since we did not have sufficient data to estimate these parameter values, we instead decided to use LHRs from the existing literature. *β*_0_ is the y intercept and is defined as the pretest odds for each etiology (Equation 3). Since this app was developed primarily for use in the ED, we decided to use the general population prevalence data for the different etiologies of syncope as our initial pretest odds for *β*_0_. This would be mean that if we did not have any other information about the patient, the statistical model would define the posttest log-odds based on the general prevalence of each etiology. The rest of the model parameters, *β_i_*, corresponded to each clinical indicator we had identified earlier. We then applied the LHRs (Equation 5) identified from the literature (after subject expert review and adjudication) to the appropriate questions. To simplify our model, we converted each variable into a binary (yes or no) question. Using the pretest odds ratio, the user input for each question, and the LHRs for each question, the app calculated a posttest log-odds ratio for each etiology of syncope and neurogenic LOC based on our logistic model. Based on these posttest probabilities calculated from posttest log-odds, the app subsequently displays a ranked order of each etiology as a DDx. The etiology with the highest probability is the most likely diagnosis.

For each type of syncope, a posttest probability *p* was calculated using logistic regression:



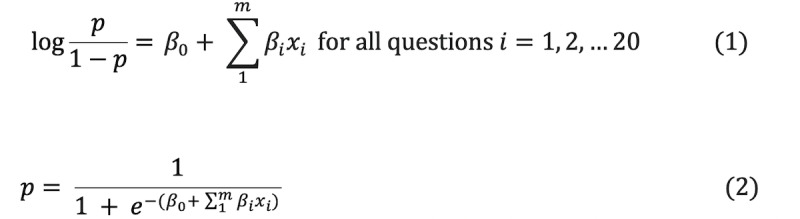



where



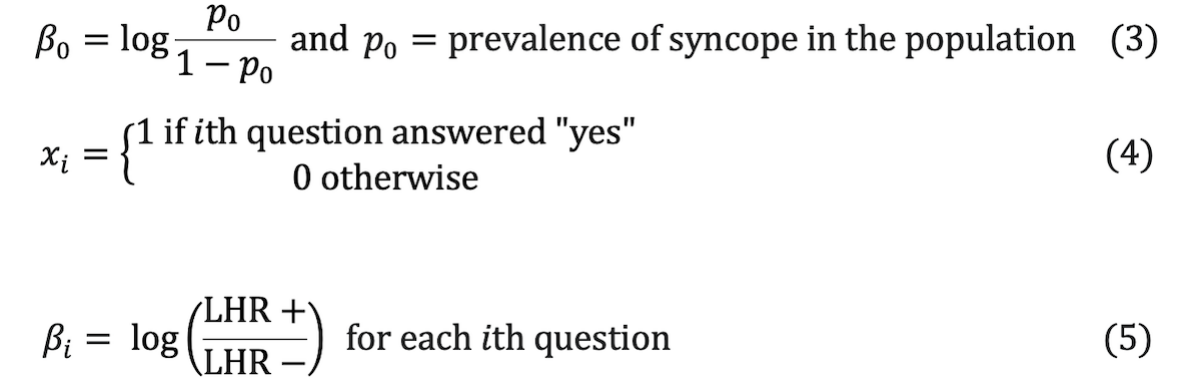



The diagnostic model was evaluated by an interdisciplinary team of experienced clinicians who assessed past cases (ie, medical chart reviews) using a web-based app developed specifically for ease of testing. The team performed a retrospective chart review of 30 patients who had presented to the ED with syncope, and compared their own diagnoses with the highest ranked differential of the statistical model. Through an iterative review and validation process, the model parameters were fine-tuned by adjusting the LHRs and the number and grouping of questions. The clinicians also identified high-risk conditions that could be missed by the diagnostic model and where a specific logic would need to be applied. The intention of this process was not to construct a perfect statistical model but rather a more pragmatic one that more closely aligns with appropriate real-world clinical practice.

The app was developed to be an adjunct to, and not a replacement for, a clinician’s evaluation experience and insight; therefore, clinicians were prompted to pick their top differential (the user-selected differential) even if it did not coincide with the highest ranked app-derived differential. This created a feedback mechanism for continued improvement of the app with data on weighting of questions as well as refinement of the mathematical formula and the parameters to the logistic regression model. Given that the Canadian Syncope Score (CSS) uses the clinician’s decision in the calculation of risk stratification, the user-selected DDx, either agreeing or disagreeing with the app-generated DDx, is used for subsequent steps in the prognosis evaluation [9]. The highest ranked app-generated DDx will be used only if the user chooses not to select a diagnosis.

### Incorporation of an Evidence-Based Clinical Risk Tool and Recommendation Development

The review of available risk score calculators focused on identifying the one easily applied and valid evidence-based clinical risk stratification tool. The review and decision-making factors included characteristics of the original study, clinical variables, and a validation study. After vetting other scoring systems, the Project MISSION team selected the CSS for risk stratification based on the available literature [9,18]. The CSS was chosen for its robust data and predictive capabilities; however, it is heavily focused on cardiogenic syncope [9]. This was felt to be appropriate clinically as true neurogenic syncope is rare (although neurogenic LOC is more often seen) [19]. Furthermore, the CSS has additive data on the timing of events in higher-risk patients.

The CSS uses a point system for available questions, two of which incorporate a general clinical assessment [9]. The answers to these questions were based on the user-selected differential when possible and otherwise defaulted to the app-derived differential. The remaining components were extracted from the user inputs from the assessment step, and a rapid classification into low (<1), intermediate (1-3), and high risk (>3) was conducted [9].

Next, we defined the scope of the recommendations by considering three different criteria: weighing diagnostic recommendations (what testing to do), disposition recommendations based on prognosis, and question-specific recommendations. These recommendations were generated based on the agreement or disagreement with app-derived and user-selected DDx. Recommendations were developed for all possible combinations of recommended and selected diagnoses.

### User-Based Design and Technical Development

In designing the user interface (UI), we relied on user-centered design principles and specific goals were considered, including to be clear and concise, only provide information as necessary, have a logical layout, and be visually appealing. Since patients suffering syncope tend to present to the ED, we began by observing physicians in the ED and the ED workflow in general to understand the needs of users and the environment in which the app would be used. This observational information combined with more input from the practicing physicians on the team was used to create a storyboard of the patient evaluation process. This storyboard was then used to construct the various screens of the app workflow, each screen representing a different step in the process. We determined the process to involve examining the patient with questions and tests, determining a DDx, assessing the risk for adverse outcomes, and, finally, providing recommendations and performing patient follow-up.

The storyboard was worked into low-fidelity wireframes to determine the appropriate elements and display the general flow of the app. This stage of the design process also included evaluating existing medical apps that provide guidelines, protocols, and risk calculators. We evaluated the ManageAnticoag and Guideline Clinical apps, both from the ACC, and Calculate by QxMD to understand the current landscape of similar apps, determine the best practices, and decide what works and what does not [20-22]. After an iterative process, these wireframes were then worked into high-fidelity mockups using Adobe Xd (Adobe Creative Cloud; Adobe Inc., San Jose, CA, USA). Using these mockups and input from the study team, we finalized the user experience (UX) and UI for the app. The MISSION Syncope app was then developed using React Native 0.60 (Facebook Open Source; Facebook Inc., Menlo Park, CA, USA), a cross-platform mobile app development framework.

While the initial wireframes were being developed, we worked on the database and web application programming interface (API) design to be used by the app to store and retrieve data. The database was designed to store the questions, the corresponding LHRs for each kind of syncope, risk stratification, and recommendation text. In addition, the database would store each syncope evaluation performed by the user, the answer to each of the questions, the app-derived differential and user-selected differential, and the final recommendation of the app. The web API was built using C# and .NET Core with the database in Microsoft SQL Server (Microsoft Corporation, Redmond, WA, USA).

Based on input from emergency medicine (EM) and other physicians, the study team determined in the early development process that the app would not require login credentials to ensure that the clinicians would be comfortable using it and the data collected would be inherently anonymous. To maintain data integrity and secure the web API, anonymous authentication was implemented using Firebase Authentication (Google Cloud Platform; Google Inc., Menlo Park, CA, USA).

Testing was an integral part of the development life cycle. There were various phases of testing, each with a specific goal. In the first phase, we focused on testing the UI and ensuring a consistent UX across devices with varying screen resolutions, screen sizes, manufacturers, and operating systems. This was performed using the App Live testing platform (BrowserStack, Mumbai, India) on real mobile phones (iOS or Android).

In the second phase of testing, the app went through an alpha-testing phase where the app was distributed using Apple’s TestFlight and Google Play’s Developer testing platforms to members of the Project MISSION team. The goal of this phase was to determine usability. This allowed the team to consider the UI/UX of the app and test it over a period on their own mobile phones and provide feedback accordingly.

After making tweaks and fixes based on this testing, participants were recruited for the final beta-testing phase. These participants were primarily physicians, fellows, and residents from EM and cardiology. This phase allowed us to open up testing to a broader audience of users who were not directly involved in the development of the app, and allowed us to gather objective feedback on usability, DDx, and performance. The beta-testers were asked to use the app for a period of several weeks, after which we conducted focus groups to obtain feedback. The focus groups were divided up based on their experiences, primarily differentiating between attending fellows or residents, and medical students. After these rounds of testing and updates to the model parameters and UI, the final version of the app was distributed through the Apple App Store and Google Play.

## Results

### DDx

The final list of questions, associations, and LHRs are provided in below (Tables 1 and 2). For variables that provided different cutoffs, the clinical lead identified the most appropriate one based on the literature and other sources (eg, a QT interval cutoff of 480 ms was used as it was part of the CSS) [9].

Some questions were included even if they did not have LHRs, given the importance in the DDx; were part of the CSS [9]; or were separately handled in the mathematical model. Since cardiac syncope (5%-21% of cases) and vasovagal syncope (21%-48%) are the highest causes of transient LOC (TLOC), most questions were defined for cardiogenic and vasovagal syncope [19]. The two questions included for orthostatic syncope had highly variable LHRs. One of the 2017 Guideline Class I recommendations is volume loading for patients with positive orthostatic vital signs, and given the overlap in the literature between positive orthostatic vitals and other causes of syncope, an additional question assessing the provider’s clinical suspicion for an orthostatic cause of syncope was added to aid in the DDx [7]. Two questions associated with neurogenic nonsyncopal episodes were included with no LHRs because neurogenic LOC is usually not true syncope. These questions purely served as a checkpoint for routing providers to a primary neurologic workup.

The logistic regression models were found to be more complete for cardiogenic and vasovagal syncope; however, we encountered issues for neurogenic LOC and orthostatic syncope due to limited studies on a DDx for these etiologies. We derived this conclusion by performing an analysis of the data collected from app usage during the alpha- and beta-testing phases. We compared the highest-ranked DDx generated by our mathematical formula with users’ selections to determine congruence with our formulas. We found that when the user determined the DDx to be cardiogenic or vasovagal, our mathematical formula derived the same result in 70% of cases. A specific logic was applied to the formula based on clinical experience. The decision was made to handle the two low-performing etiologies, orthostatic and neurogenic LOC, by applying artificial weighting to move these etiologies to the top of the differential if all questions for each type were answered with yes and by providing targeted question-specific recommendations. For example, the MISSION Syncope app recommends volume loading when a patient is orthostatic, but the app-derived or user-selected differential can be cardiogenic or vasovagal, respectively [7]. For neurogenic LOC, weighting was purely based on clinical experience, with no reference to a discrete LHR, because no consistent LHR exists in the literature that would mathematically rank this etiology to the top of the differential. In addition, since the neurogenic risk to the patient (prognosis) was not addressed by the CSS, a deliberate mention of concerning features and additive recommendations were deemed important [9].

**Table 1:** Final assessment questions, with LHRs^a^ for vasovagal syncope.

Question	Reference	LHR+	LHR–
Is the patient less than or equal to 35 years of age?	[16]	7.29	0.30
Does the patient have a history of heart disease (atrial fibrillation/flutter, ventricular tachycardia, heart block, heart failure, stable ischemic heart disease, valvular heart disease)?^b^	[15,16,23]	0.072	1.82
Did the syncopal episode occur in the context of any of the following: warm or crowded place, prolonged standing, fear, emotion, pain, or using the toilet?^b^	[16]	8.85	0.498
Was the syncopal episode associated with chest pain?	[23,24]	NULL^c^	NULL^c^
Was the syncopal episode associated with palpitations?	[13,15,16,23-25]	NULL^c^	NULL^c^
Was the syncopal episode associated with exertion?	[13,15]	NULL^c^	NULL^c^
Was the syncopal episode associated with position change?^d^	—	NULL^c^	NULL^c^
Was the syncopal episode associated with hypoxia?	[16]	0.104	1.08
Was the syncopal episode associated with nausea, vomiting, or a warm/flushed feeling?	[13,15,16,23-25]	5.10	0.552
Does the patient describe any of the following: severe headache, focal neurologic deficit, or postictal state?^e^	[16]	NULL^c^	NULL^c^
Were there convulsions witnessed associated with the syncope?^d^	—	NULL^c^	NULL^c^
Is there a new murmur on exam?^d^	—	NULL^c^	NULL^c^
Is the resting SBP^f^ <90 mmHg or >180 mmHg?	[16]	NULL^c^	NULL^c^
Were orthostatic vitals positive (>20 mmHg drop in SBP or >30 beats per minute increase in heart rate)?^g^	—	NULL^c^	NULL^c^
Do you think orthostasis is the cause for syncope?^g^	—	NULL^c^	NULL^c^
Were there any new focal neurologic deficits on physical exam?^e^	—	NULL^c^	NULL^c^
Is the QRS axis abnormal (<–30 degrees or >100 degrees)?^b^	—	NULL^c^	NULL^c^
Is the QRS duration prolonged (>120 ms)?^b^	—	NULL^c^	NULL^c^
Is the corrected QT interval prolonged (>480 ms)?^b^	—	NULL^c^	NULL^c^
Is the troponin elevated (high-sensitivity cardiac troponin *t*>14 ng/L)?^b^	[26]	NULL^c^	NULL^c^

^a^LHR: likelihood ratio.

^b^Input for the Canadian Syncope Score (CSS).

^c^Ratios not found in the literature.

^d^Included to prompt additional considerations.

^e^Artificially weighted for neurogenic loss of consciousness (LOC).

^f^SBP: systolic blood pressure.

^g^Artificially weighted for orthostatic syncope.

**Table 2:** Final assessment questions, with LHRs^a^ for cardiogenic syncope.

Question	Reference	LHR+		LHR–
Is the patient less than or equal to 35 years of age?	[16]	0.13	3.24	
Does the patient have a history of heart disease (atrial fibrillation/flutter, ventricular tachycardia, heart block, heart failure, stable ischemic heart disease, valvular heart disease)?^b^	[15,16,23]	2.93	0.74	
Did the syncopal episode occur in the context of any of the following: warm or crowded place, prolonged standing, fear, emotion, pain, or using the toilet?^b^	[16]	0.167	1.43	
Was the syncopal episode associated with chest pain?	[23,24]	4.25	0.881	
Was the syncopal episode associated with palpitations?	[13,15,16,23-25]	3.78	0.853	
Was the syncopal episode associated with exertion?	[13,15]	4.36	0.896	
Was the syncopal episode associated with position change?^c^	—	NA^d^	NA	
Was the syncopal episode associated with hypoxia?	[16]	3.74	0.94	
Was the syncopal episode associated with nausea, vomiting, or a warm/flushed feeling?	[13,15,16,23-25]	0.354	1.38	
Does the patient describe any of the following: severe headache, focal neurologic deficit, or postictal state?^e^	[16]	0.170	1.21	
Were there convulsions witnessed associated with the syncope?^c^	—	NULL^f^	NULL^f^	
Is there a new murmur on exam?^c^	—	NULL^f^	NULL^f^	
Is the resting SBP^g^ <90 mmHg or >180 mmHg?	[16]	5.88	0.894	
Were orthostatic vitals positive (>20 mmHg drop in SBP or >30 beats per minute increase in heart rate)?^h^	—	NULL^f^	NULL^f^	
Do you think orthostasis is the cause for syncope?^h^	—	NULL^f^	NULL^f^	
Were there any new focal neurologic deficits on physical exam?^e^	—	NULL^f^	NULL^f^	
Is the QRS axis abnormal (<–30 degrees or >100 degrees)?^b^	—	NULL^f^	NULL^f^	
Is the QRS duration prolonged (>120 ms)?^b^	—	NULL^f^	NULL^f^	
Is the corrected QT interval prolonged (>480 ms)?^b^	—	NULL^f^	NULL^f^	
Is the troponin elevated (high-sensitivity cardiac troponin *t*>14 ng/L)?^b^	[26]	1.98	0.534	

^a^LHR: likelihood ratio.

^b^Input for the Canadian Syncope Score (CSS).

^c^Included to prompt additional considerations.

^d^NA: not available.

^e^Artificially weighted for neurogenic loss of consciousness (LOC).

^f^Ratios not found in the literature.

^g^SBP: systolic blood pressure.

^h^Artificially weighted for orthostatic syncope.

### Incorporation of an Evidence-Based Clinical Risk Tool and Recommendation Development

Based on the MISSION Syncope OptimalCare Pathway, recommendations included testing recommendations and disposition. Considering the lack of space on a mobile screen for large amounts of text, the Project MISSION team decided to categorize these recommendations into primary, secondary, and question specific. Primary recommendations were based on the user-selected DDx or that generated by the app if a user did not pick a diagnosis. The secondary recommendations would only be displayed if the app-generated DDx was discordant with the user-selected DDx. The question-specific recommendations referred to considerations specifically for orthostasis and neurogenic LOC if app users answered positively to only one of the questions associated with the etiology. Finally, based on the CSS classification, the implication of the risk score was provided as either discharge from the ED with outpatient workup (low risk), short-term observation of 6 hours or less (intermediate risk), or longer-term observation and admission (high risk).

### User-Based Design and Technical Development

The app provides a presentation layer with limited logic and data storage and is designed to work online, with interactions processed on the backend web API and stored in the server database. We collected all the information anonymously and recorded answers to all the assessment questions, the highest-ranked app-generated DDx, the user-selected DDx, and the CSS, allowing us to gather valuable information about syncope cases and to validate our model.

After input from frontline clinicians, we designed four screens that would be part of the main app workflow and reflect the steps in a real-world patient evaluation: Assessment, Differential Diagnosis, Risk Stratification, and Recommendations. See Multimedia Appendices 1 and 2 for the app installation guide and app assessment examples, respectively. The Assessment screen in the initial design consisted of a card deck–like UI, with each question on a separate card, a UI design most commonly seen in trivia apps. Our beta-testing focus group almost unanimously recommended against this design and instead recommended displaying the questions in list format. In addition to the aforementioned screens, based on user feedback, we determined that a home screen would be needed to have a place for the user to start new evaluations and view all the evaluations they have performed in the past. The list of evaluations included information about risks and the DDx of each. Based on the recommendations from the beta-testing focus group, we also included onboarding screens to the app. These are shown to the user when they start the app for the first time, and walk them through the purpose and usage of the app. In addition to the usability of the app, the beta- and alpha-testing phases allowed us to judge the consistency of the app diagnosis and recommendation.

## Discussion

### Principal Findings

Most CDS tools have a narrow scope of utility; they either are risk score calculators (eg, MDCalc) or provide information that might not be effectively specific (eg, ManageAnticoag) [20,27]. These latter apps often have a comprehensive list of variables, which limits the capability of providing specific, actionable recommendations and complicates decision making. The existing CDS tools for syncope are limited and only provide a risk score without subsequent recommendations or diagnostic support. Mobile apps also suffer from such a narrow scope, either focusing on patient education/engagement or translating risk score calculators to a mobile platform. Most publications on mobile apps fall into one of three categories: (1) proposal or initial development of a medical mobile app, (2) specific considerations in the development of mobile apps (eg, security or usability), and (3) position papers to improve standardization or evaluate the efficacy of apps. Our study falls into the first category. However, most of these apps are designed for patient engagement and education or risk calculators with embedded guideline recommendations. Our app development study is distinct in that provides prognostic information based on the validated CSS, an evidence-based approach to the DDx, and recommendations consistent with ACC/AHA guidelines [7,9]. There are also limited CDS tools for the diagnosis and management of syncope specifically. Several key learning points from this development process include (1) handling gaps in the literature and lack of a pre-existing study with such a model, (2) clearly and deliberately defining the scope of the app, (3) finding a balance between perfection and usability, and (4) dealing with technical challenges inherent to building a medical app. It is imperative to consciously identify and accept the potential of uncertainty in this type of project, as gaps exist in published research on patients presenting with syncope. We felt it most appropriate to mirror a clinical approach to these gaps by using adjunct variables, applying values to variables that are consistent with clinical experience, and deciding alternative approaches to addressing high-risk variables. This cannot be attempted in isolation and needs to be thoroughly vetted and tested within an interdisciplinary team with clinical, statistical, and technical expertise. As such, the differentiating factor of such a team is successfully combining the various domains of knowledge into a cohesive approach.

Defining the scope of an app is a necessary part of any software implementation and requires a dedicated focus during the ideation phase of the app design. Otherwise, there is a risk of building extraneous details into an app that will not be clinically useful and will be distracting to the user. The aim was to create an app that is clear, concise, and consistent, while providing the user with specific and actionable information and not complicating the UX with cluttered and generic information. With the MISSON Syncope app, we took a more holistic implementation approach with a focused set of recommendations. This app benefited from having a well-developed MISSION Syncope OptimalCare Pathway. A care pathway is the synthesis of guideline recommendations into an optimal pattern of practice considering complex clinical features that are part of the DDx and workup. Therefore, in similar software implementation, development of a care pathway should be a critical first step in the process. The pathway can guide what questions would be asked and what recommendations would be provided; without this, the app can become a series of disjointed questions addressing guideline recommendations that could be, at times, discordant with one another.

Successful app implementation requires a deliberate pursuit of a practical formula instead of striving for a perfect mathematical model. Creation of a nuanced, comprehensive formula results in a large number of variables, and although more extensive and possibly more accurate, it might not be practical for use as a mobile app. Additionally, since a perfect formula is untenable, given the current data and knowledge, potential incorrect assessments without acknowledging uncertainties and probabilities may provoke users to devalue the app and result in less acceptance. Therefore, a natural design point would be deciding between a perfect mathematical formula and a practical formula. Additionally, the formula was limited by a lack of study data and the LHRs available in the current literature, especially for orthostatic and neurogenic syncope. Importantly, the data collected by the app can provide useful information about filling gaps in the evaluation of syncope and guide future modifications.

Finally, in developing the app, we had to make a few deliberate decisions about the technical buildout. Deciding the supported screen sizes was especially challenging when building a cross-platform app that runs on both iOS and Android devices. With limited resources and a small development team, we decided to use the BrowserStack App Live testing platform to test the app on numerous real devices. Another challenge was deciding whether a user should be required to log into the app. To promote easy adoption and to secure the backend API, we developed an anonymous authentication system using the Firebase (Google LLC) mobile platform. This also helped in making our data collection inherently anonymous. One challenge that does not have a clear solution is determining the veracity of each evaluation—in other words, determining which evaluations are genuine and which are hypothetical scenarios that users might be entering to test out the app. One solution can be to disregard the first few evaluations for each user; another could be to allow the user to flag such hypothetical scenarios. Future versions of the app could incorporate technical changes based on use.

App development is an iterative process with opportunities for continuous improvement. Having gone through the development process, our team has become more informed about how to apply app development to other disease states and develop similar clinical support tools.

### Limitations

The limitations of this study included a lack of app evaluation by a larger number of users. The app will likely benefit from evaluation with input from a larger number of syncope cases by comparing the physicians’ DDx and app-derived DDx for these cases. To test and improve our statistical model, we provide the user with a ranked order of differentials and determine the accuracy of the app by comparing the app-generated DDx to the user-selected DDx, thus creating a feedback loop for continuous improvement. Another limitation is that although we set up alpha- and beta-testing groups, we did not have extensive user feedback on how well the UI works and where it can be improved. Future work would include conducting a comprehensive retrospective chart review study, where we will evaluate a larger number of syncope cases that are presented in the ED against the DDx, recommendation, and risk stratification provided by the app. In addition, we plan to set up a process to continuously update the model parameters based on new studies and LHRs in continued literature reviews.

### Conclusion

The MISSION Syncope app development integrated the current literature and clinical expertise to provide an evidence-based DDx, a prognosis using a validated scoring system, and recommendations based on clinical guidelines. This app demonstrates the importance of using research literature in the development of a CDS tool and applying clinical experience to fill the gaps in available research. It is essential for a successful app to be deliberate in pursuing a practical clinical model instead of striving for a perfect mathematical model, given available published evidence. This hybrid methodology can be applied to similar CDS tool development.

## Appendix

Multimedia Appendix 1 QR code and app download instructions.

Multimedia Appendix 2 Sample screenshots of the mobile app.

## Abbreviations

ACC: American College of Cardiology
ACEP: American College of Emergency Physicians
ACS: acute coronary syndrome
AHA: American Heart Association
API: application programming interface
CAD: coronary artery disease
CDS: clinical decision support
CHF: congestive heart failure
CPG: clinical practice guideline
CSS: Canadian Syncope Score
DDx: differential diagnosis
EBP: evidence-based practice
ECG: electrocardiogram
ED: emergency department
EM: emergency medicine
HRS: Heart Rhythm Society
LHR: likelihood ratio
LOC: loss of consciousness
LVOT: left ventricular outflow tract
MISSION: Multilevel Implementation Strategy for Syncope optImal care thrOugh eNgagement
PCP: primary care provider
PO: per os
SAEM: Society for Academic Emergency Medicine
SBP: systolic blood pressure
SDM: shared decision making
TLOC: transient loss of consciousness
UI: user interface
UX: user experience
