# Supplementary material for: Developing and Demonstrating the Viability and Availability of the Multilevel Implementation Strategy for Syncope Optimal Care Through Engagement (MISSION) Syncope App: Evidence-Based Clinical Decision Support Tool
Source: J Med Internet Res. 2021 Nov 16;23(11):e25192. doi: 10.2196/25192 (PMC8663445; doi:10.2196/25192)
Supplement: Multimedia Appendix 1 [file jmir_v23i11e25192_app1.pdf]

APPENDIX
MISSION Syncope: Installation Guide

Steps to install MISSION Syncope:

1. Open this link from your phone: http://onelink.to/vs24bf
2. Click on install after the link opens your phone’s app store (App Store or Play Store)
3. If the above link does not work. Search for MISSION Syncope in your phone’s app store and click on the install button.

In addition, here is a QR code:

Open your phone’s camera app and point the camera at the QR code.

1. Once prompted, follow the link to your phone’s app store.
2. Click on the install button to install MISSION Syncope.
