# Supplementary material for: Developing and Demonstrating the Viability and Availability of the Multilevel Implementation Strategy for Syncope Optimal Care Through Engagement (MISSION) Syncope App: Evidence-Based Clinical Decision Support Tool
Source: J Med Internet Res. 2021 Nov 16;23(11):e25192. doi: 10.2196/25192 (PMC8663445; doi:10.2196/25192)

Easy Patient Assessment

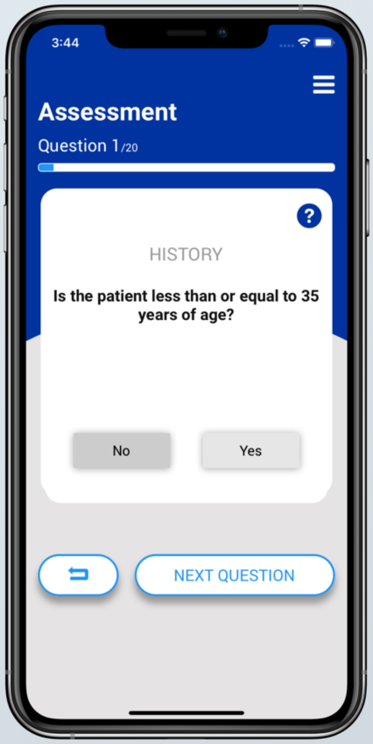

Ranked Differential

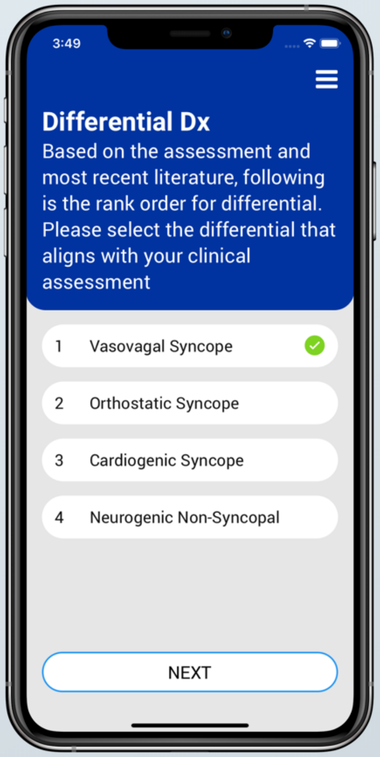

Next steps. Recommendation.

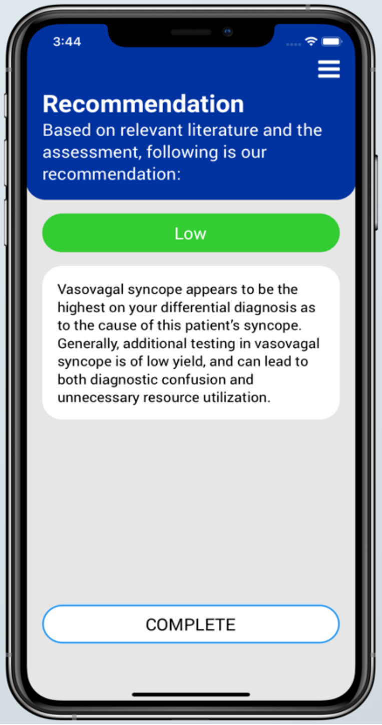

Risk Assessment

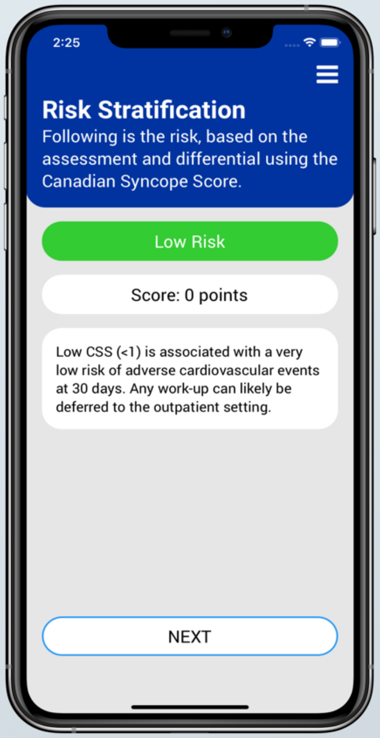

Supplement: Multimedia Appendix 2 [file jmir_v23i11e25192_app2.pdf]
